# Activation of cytosolic DNA sensing in NCM460 intestinal epithelial cells induces antiviral factors that restrict HIV infection in macrophages

**DOI:** 10.3389/fimmu.2026.1919977

**Published:** 2026-09-30

**Authors:** Safah Majid, Shazheb Khan, Xu Wang, Raheeb Khan, Qian-Hao Xiao, Yuan Rong, Zhi Wei, Qing-Sheng Li, Wen-Zhe Ho

**Affiliations:** 1 Department of Pathology and Laboratory Medicine, Temple University Lewis Katz School of Medicine, Philadelphia, PA, United States; 2 Department of Computer Science, Ying Wu College of Computing, New Jersey Institute of Technology, Newark, NJ, United States; 3 HIV Cure and Viral Diseases Center, The Wistar Institute, Philadelphia, PA, United States

**Keywords:** CC chemokines, DNA sensors, HIV, IFN-β/λ, poly (dA:dT)

## Abstract

**Introduction:**

The gastrointestinal tract is an important site of HIV persistence and contains susceptible target cells, including macrophages. Crosstalk between intestinal epithelial cells and macrophages may contribute to mucosal innate defense.

**Methods:**

NCM460 human intestinal epithelial cells were transfected with poly(dA). Expression and production of interferons, interferon-stimulated genes, and CC chemokines were assessed by quantitative RT-PCR, western blotting, and ELISA. Primary human macrophages were treated with conditioned supernatants from NCM460 cells, and HIV infection was assessed using cytopathic effects, luciferase activity, and HIV Gag RNA expression.

**Results:**

Poly(dA) induced IFN-β, IFN-λ1, IFN-λ2/3, ISG15, viperin, OAS1, RANTES, and MIP-1α in NCM460 cells. Supernatants from poly(dA)-stimulated NCM460 cells induced GBP5, viperin, and MX2 in primary macrophages and significantly inhibited HIV infection.

**Discussion:**

Activation of cytosolic DNA sensing in intestinal epithelial cells induces soluble antiviral factors that enhance innate antiviral responses in macrophages and may contribute to mucosal defense against HIV.

## 1 Introduction

The gastrointestinal (GI) tract is an important site of HIV establishment and persistence, as the mucosa contains abundant HIV target cells, including macrophages and activated CD4^+^ T lymphocytes. GI-resident macrophages constitute one of the body’s largest populations of mononuclear phagocytes (1–3). As a primary physical barrier, intestinal epithelial cells (IECs) maintain gut integrity and regulate mucosal immunity through innate immune sensors that recognize microbial components (4). IECs express various pattern-recognition receptors (PRRs), including cytosolic DNA sensors, RIG-I-like receptors, and Toll-like receptors (TLRs) (5, 6). Recognition of cytosolic DNA can induce interferons (IFNs) and downstream interferon-stimulated genes (ISGs) (7). Poly(dA:dT), a synthetic B-form double-stranded DNA analog, is commonly used to activate cytosolic DNA sensing pathways experimentally (8).

Cytosolic DNA sensing plays an important role in antiviral innate immunity (9, 10). Cyclic GMP-AMP synthase (cGAS) is a major DNA sensor that activates the STING-TBK1 pathway to promote IFN production (11–14). RNA–DNA hybrids can also induce type I IFN responses through the cGAS–STING pathway (15–17). cGAS has been implicated in the recognition of HIV reverse transcription products and induction of antiviral responses (18–22). Although DNA sensing has been studied primarily in immune cells, non-immune cells, including epithelial cells, also possess functional DNA sensing machinery (8, 10, 23–25).

Given the importance of the GI tract in HIV persistence, understanding crosstalk between IECs and macrophages may provide insight into mucosal antiviral immunity. Although IECs generally do not support productive HIV infection (26), they can produce chemokines, cytokines, and antimicrobial factors that modulate mucosal immune responses (27, 28). Previous studies demonstrated that TLR3 activation in IECs induces soluble factors that suppress HIV infection in macrophages (29), while direct activation of cytosolic DNA sensing in primary human macrophages restricts HIV infection (30). Building on these observations, we investigated whether poly(dA:dT)-induced cytosolic DNA sensing in NCM460 intestinal epithelial cells generates soluble antiviral factors capable of enhancing antiviral responses and restricting HIV infection in primary human macrophages.

## 2 Materials and methods

### 2.1 Reagents

Cell culture plasticware were obtained from Corning (Corning, NY, USA). All the culture reagents were purchased from Gibco (Grand Island, NY, USA). Rabbit antibodies against OAS-1, Viperin, ISG15, and GAPDH were purchased from Cell Signaling Technology (Danvers, MA, USA). The Lyovec and poly (dA:dT) were purchased from InvivoGen (San Diego, CA, USA). LyoVec was used to transfect NCM460 cells with poly(dA:dT), a ligand for cytosolic DNA sensors.

### 2.2 Cell culture

We used the human intestinal epithelial cell line (NCM460) that was originally derived from normal and non-transformed human colonic mucosa (29, 31). Unlike commonly used cancer-derived cell lines such as Caco-2 and HT-29, NCM460 cells retain a normal epithelial phenotype and a diploid karyotype, representing a more physiologically relevant model of the primary intestinal epithelial barrier. Cells were cultured at 37 °C with 5% CO2 and 100% humidity and the culture medium was changed every 48-72h. For preparation of NCM460 culture supernatants, cells were transfected with or without poly (dA:dT) at different concentrations (0.01, 0.1, or 1μg/mL) for 6h, washed three times with plan RPMI, and then cultured for 18h. The cell-free supernatants (SN) were then collected and used as NCM460 SN.

We used the de-identified purified human peripheral blood monocytes that were purchased from Human Immunology Core and the Division of Transfusion Medicine and Therapeutic Pathology at the University of Pennsylvania Perelman School of Medicine (Philadelphia, PA, USA). Monocytes were cultured in 48-well plates (Corning CELLBIND Surface, Kennebunk, ME, USA) at 37 °C and 5% CO2 in Dulbecco’s modified Eagle’s culture medium containing 10% FBS, 2mM L-glutamine, 50 units/ml penicillin, and 50μg/ml streptomycin. Monocytes were differentiated into macrophages after *in vitro* culture for seven days. We used 7-day-cultured macrophages for all the experiments in this study.

### 2.3 MTS assay

The cytotoxic effect of poly (dA:dT) on NCM460 cells was evaluated using the MTS (3-(4, 5-dimethylthiazol-2-yl)-5-(3-carboxymethoxyphenyl)-2-(4-sulfophenyl)-2H-tetrazolium, inner salt) assay. NCM460 cells (2 × 10^4 cells/well) were plated in 96-well round-bottom plates and treated with different concentrations of poly (dA:dT) (0, 0.1, 0.5, 1, and 5 μg/ml) or 10% DMSO (positive control) for 72 h. The cells were then incubated with CellTiter 96^®^ AQueous One Solution Reagent (Promega, Madison, WI) containing MTS and phenazine ethosulfate for 4 h at 37 °C. The absorbance at 490 nm was measured using a plate reader (SpectraMax i3, Molecular Devices, Sunnyvale, CA, USA). As shown in **Supplementary Figure 1**, poly (dA:dT) at the tested dosages (0.1, 0.5, 1, and 5μg/ml) had little cytotoxic effect on NCM460 cells.

### 2.4 Quantitative RT-PCR

NCM460 cells were transfected with or without poly(dA:dT) at different concentrations (0.01, 0.1, and 1 μg/mL) and collected at 12 and 24 h post-transfection. Seven-day cultured macrophages were treated with or without NCM460 cell supernatant (SN; 0.2%, 2%, or 20%) for 24 h. Total cellular RNA was extracted using TRI Reagent (Sigma-Aldrich, St. Louis, MO) and reverse-transcribed into cDNA using random primers, dNTPs, recombinant RNasin ribonuclease inhibitor, and AMV reverse transcriptase (Promega, Madison, WI). Quantitative RT-PCR was performed using PowerUp SYBR Green Master Mix and a QuantStudio 3 system (Applied Biosystems, Waltham, MA, USA) with primers specific for the indicated target genes. GAPDH mRNA was used as an endogenous reference for normalization of target gene expression (32, 33). The primer information for all quantitative RT-PCR assay is listed in the **Supplementary Table 1**.

### 2.5 Western blot

Western blotting was performed to determine the protein expression of interferon-stimulated genes (ISG15, OAS-1, and Viperin). Cells were lysed in RIPA buffer supplemented with protease/phosphatase inhibitors (Sigma-Aldrich, St. Louis, MO, USA). Proteins from culture supernatants were extracted using TCA/acetone precipitation. Briefly, 0.5 mL of culture supernatant was mixed with 0.5 mL of 20% TCA and incubated at −20 °C for 30 min, followed by centrifugation at 11,500 rpm for 15 min at 4 °C. After three washes with 1 mL of ice-cold acetone, the protein pellet was resuspended in Western blot lysis buffer. Protein concentrations were determined using a bicinchoninic acid (BCA) assay (ChemCruz, Dallas, TX, USA). Blots were incubated with primary antibodies in 5% non-fat milk overnight at 4 °C, washed with PBS containing 0.1% Tween-20 (PBST), and incubated with horseradish peroxidase-conjugated secondary antibodies for 1 h at room temperature. After washing with PBST, protein signals were detected using enhanced chemiluminescence (Amersham, Bucks, UK) and imaged using an iBright 1500 Imaging System (Invitrogen, CA, USA) (34, 35).

### 2.6 HIV infection

HIV Bal was obtained from the NIH AIDS Reagent Program (Bethesda, MD), and HIV Bal expressing a luciferase reporter (HIV Bal-eLuc) was provided by Dr. Won-Bin Young (36). Monocyte-derived macrophages were pretreated for 24 h with or without NCM460 cell supernatant (SN; 0.2%, 2%, or 20%) and then infected with HIV Bal or HIV Bal-eLuc. Following infection, cells were washed three times with DMEM to remove unbound virus, and culture supernatants were collected every three days. HIV infection was assessed on day 6 after infection by luciferase activity and HIV Gag/LTR RNA expression (37, 38).

### 2.7 ELISA

IFN-β, IFN-λ, MIP-1α, and RANTES protein levels in NCM460 SN were measured by ELISA (IFN-β: Invitrogen; IFN-λ1/3, IFN- λ2, MIP-1α, and RANTES: R&D Systems Inc., Minneapolis, MN, USA). The commercially available ELISA kit (R&D Systems) detects IFN-λ1/3 but does not distinguish between IFN-λ1 and IFN-λ3. Assays were performed according to the manufacturer’s instructions.

### 2.8 Statistical analysis

Data are expressed as the mean ± standard deviation (SD). Statistical significance was determined using one-way/two-way ANOVA or multiple t-tests with GraphPad Prism software (GraphPad Software, La Jolla, CA, USA). P < 0.05, P < 0.01, P < 0.001, and P < 0.0001 were considered statistically significant.

## 3 Results

### 3.1 Upregulation of DNA sensors by poly(dA:dT) treatment

Cytosolic DNA sensors play important roles in the recognition of intracellular DNA and initiation of innate immune responses. To assess the response of NCM460 cells to cytosolic DNA stimulation, we examined the expression of key DNA sensing components following poly(dA:dT) transfection. As shown in **Figure 1A**, poly(dA:dT) significantly increased the mRNA expression of cGAS, IFI16, and STING, indicating that cytosolic DNA stimulation enhances the expression of DNA sensing components in NCM460 cells.

**Figure 1:**
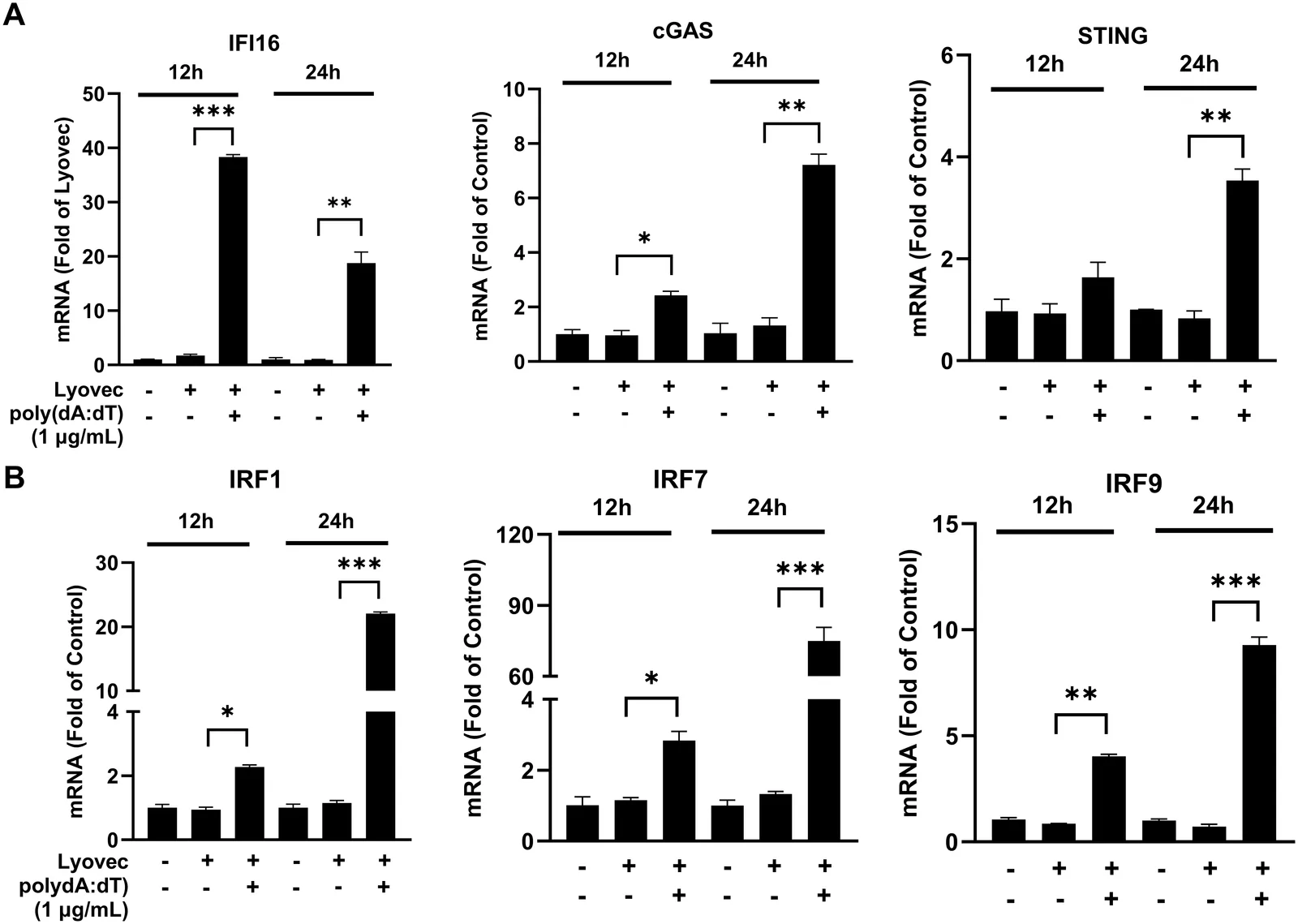
Poly (dA:dT) treatment of IECs induces DNA sensors (IFI16, cGAS), STING, and IRF1/7/9. **(A)** NCM460 cells were transfected with 1 µg/ml of poly (dA:dT) for 12 h and 24 h. Total cellular RNA was collected and subjected to quantitative RT-PCR for the expression of cGAS, IFI16, and STING **(A)**, IRF1, IRF7, and IRF9 **(B)**. Data shown are representative of three independent experiments with NCM460 cells. Asterisks indicate that the differences between the indicated groups are statistically significant (*p < 0.05, **p < 0.01, ***p < 0.001).

### 3.2 Induction of IFN regulatory factors by poly(dA:dT)

IFN regulatory factors (IRFs), including IRF1, IRF3, IRF7, and IRF9, are important mediators of interferon-driven immune responses (39, 40). We therefore examined the effect of poly(dA:dT) stimulation on the expression of these factors in NCM460 cells. Poly(dA:dT) treatment did not significantly alter IRF3 mRNA expression (data not shown). Because IRF3 activity is primarily regulated through phosphorylation and nuclear translocation, unchanged transcript levels do not exclude its potential involvement. In contrast, the expression of IRF1, IRF7, and IRF9 increased significantly at both 12 and 24 h post-transfection. (**Figure 1B**). Further studies assessing IRF3 phosphorylation and nuclear translocation will be required to determine its functional activation in response to poly(dA:dT).

### 3.3 Poly(dA:dT) activates IFNs and antiviral ISGs

Because cytosolic DNA sensing can induce innate immune responses through interferon pathways (13, 21, 41, 42), we next examined whether poly(dA:dT) stimulation induces IFN production in NCM460 cells. Poly(dA:dT) transfection significantly increased the expression of type I (IFN-β) and type III (IFN-λ) interferons at both the mRNA and protein levels in a dose-dependent manner (**Figures 2A, B**). In parallel, poly(dA:dT) also induced dose-dependent increases in the mRNA expression of the antiviral ISGs ISG15, OAS-1, and Viperin (**Figure 3**).

**Figure 2:**
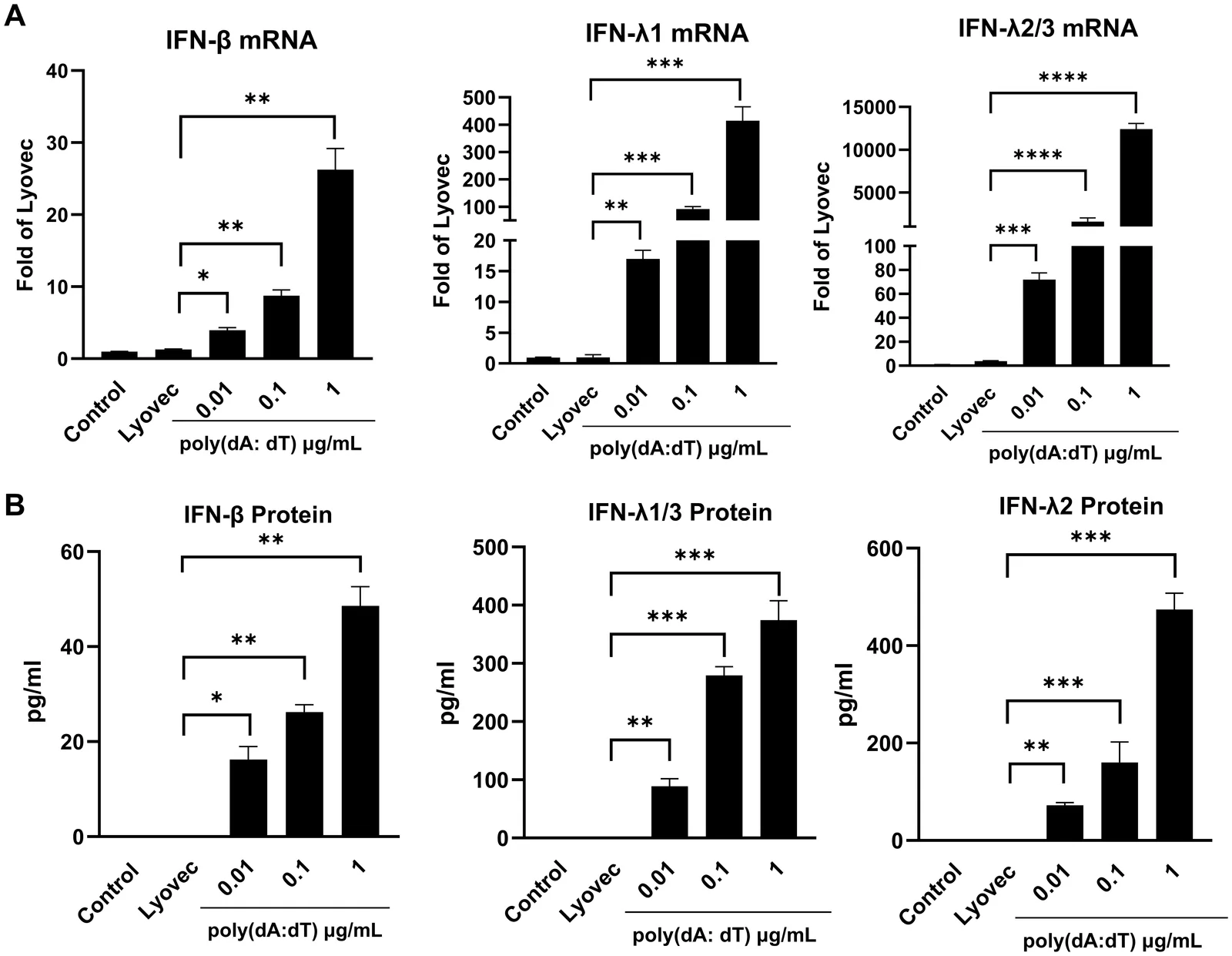
Effect of cytosolic DNA sensor activation by poly (dA:dT) on type I and type III IFN expression. **(A)** NCM460 cells were transfected with the indicated doses of poly (dA:dT) for 24 h prior to quantitative RT-PCR analysis of the transcriptional upregulation of IFN-β, IFN-λ1, and IFN-λ2/3 genes. **(B)** IFN protein levels in culture supernatant were quantified by ELISA. The experimental findings represent consistent patterns observed across three biological replicates, representative of three independent experiments. Statistical significance between experimental groups is denoted as follows: *p < 0.05, **p < 0.01, ***p < 0.001.

**Figure 3:**
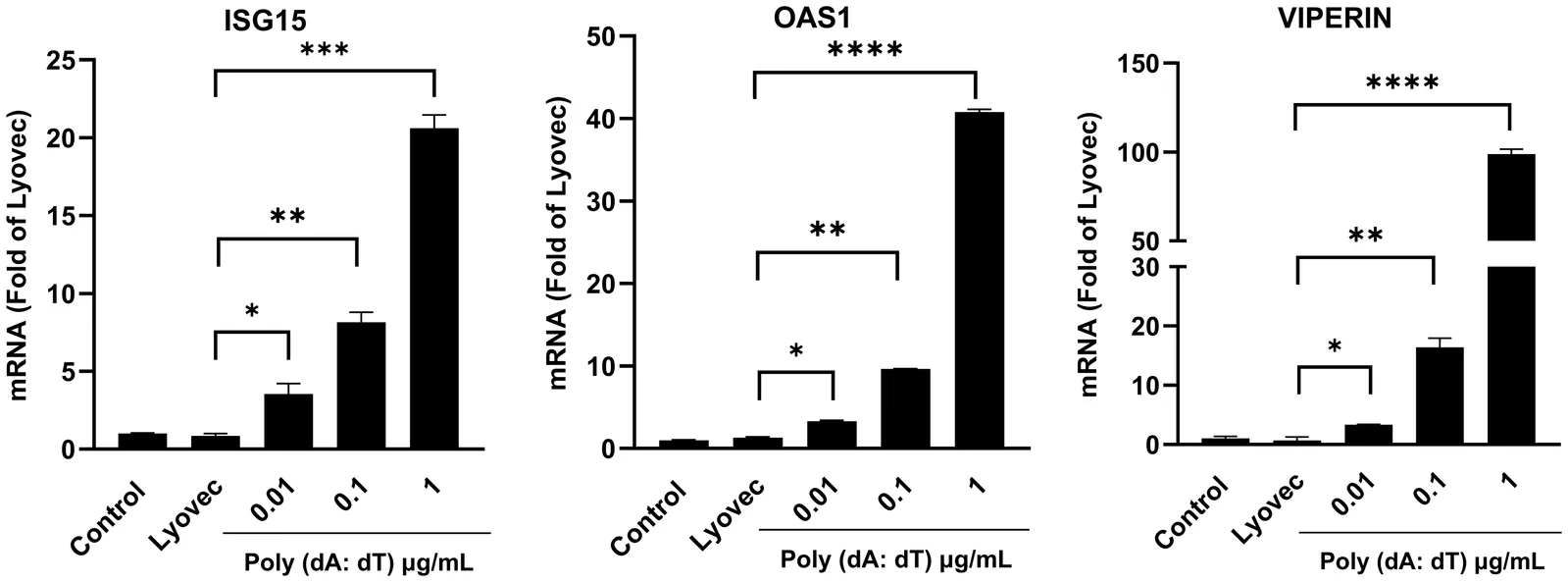
Poly (dA:dT) induces ISGs in IECs. NCM460 cells were transfected with indicated concentrations of poly (dA:dT) for 24 h. Total cellular RNA was extracted and subjected to quantitative RT-PCR for the expression of ISG15, OAS-1 and viperin. Data shown are representative of three independent experiments. Asterisks indicate that the differences between the indicated groups are statistically significant (*p < 0.05, **p < 0.01, ***p < 0.001, ****p < 0.0001).

### 3.4 Upregulation of CC chemokines by poly(dA:dT) stimulation

Certain CC chemokines, including RANTES and MIP-1α/β, are natural ligands of CCR5 and can inhibit HIV entry by competing for CCR5 binding (42–45). To determine whether cytosolic DNA stimulation induces these antiviral factors, we examined CC chemokine expression in NCM460 cells following poly(dA:dT) transfection. As shown in **Figure 4**, poly(dA:dT) significantly increased MIP-1α and RANTES expression at both the mRNA (**Figure 4A**) and secreted protein (**Figure 4B**) levels in a dose-dependent manner. 

**Figure 4:**
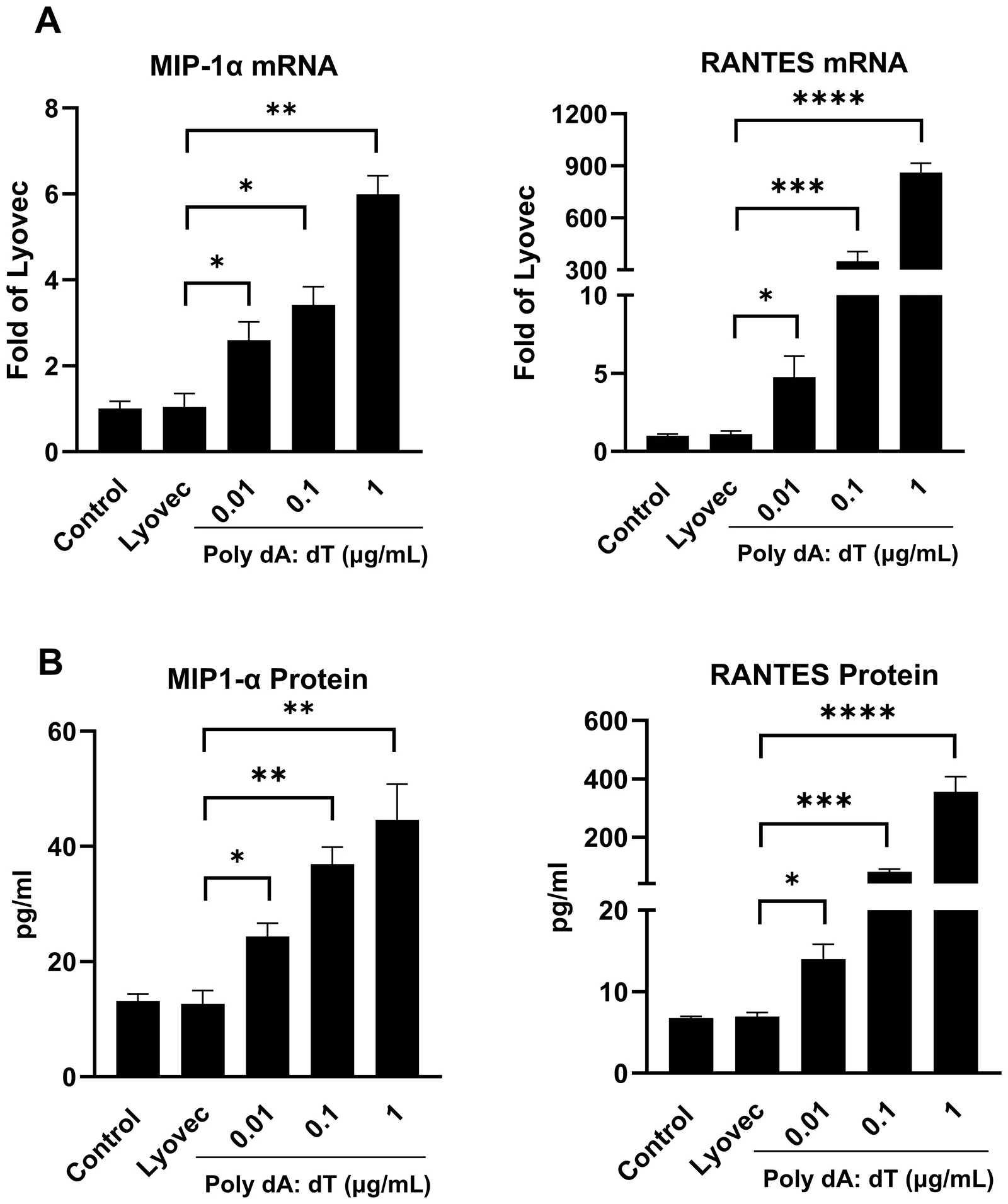
Activation of DNA sensors induces CC chemokines. NCM460 cells were transfected with poly (dA:dT) at indicated concentrations for 24 hr. **(A)** Total cellular RNA was extracted and subjected to quantitative RT-PCR for the expression of CC chemokines (MIP-1α and RANTES). **(B)** Secreted protein levels in culture supernatant for MIP-1α and RANTES were measured by ELISA. Data shown are representative of three independent experiments. Asterisks indicate that the differences between the indicated groups are statistically significant (*p < 0.05, **p < 0.01, ***p < 0.001, ****p < 0.0001).

### 3.5 Induction of antiviral factors and HIV restriction in macrophages by activated IEC supernatants

Building on our previous findings that direct cytosolic DNA sensor activation restricts HIV infection in macrophages (30), we investigated whether poly(dA:dT)-stimulated NCM460 cells could induce antiviral responses in macrophages through soluble factors. Conditioned supernatant (SN) from poly(dA:dT)-transfected NCM460 cells was applied to primary human macrophages. Treatment with SN significantly increased the expression of the antiviral ISGs GBP5, Viperin, and MX2 at both the mRNA (**Figure 5A**) and protein (**Figure 5B**) levels. Macrophages pretreated with SN from poly(dA:dT)-stimulated NCM460 cells also exhibited reduced HIV-induced cytopathic effects, including giant syncytia formation (**Figure 6A**), and significantly decreased HIV infection as measured by luciferase activity (**Figure 6B**). Consistently, HIV Gag RNA levels were reduced in both macrophages and culture supernatants (**Figures 6C, D**). The extent of HIV inhibition increased with the amount of SN applied, supporting a dose-dependent antiviral effect of soluble factors released from poly(dA:dT)-stimulated NCM460 cells.

**Figure 5:**
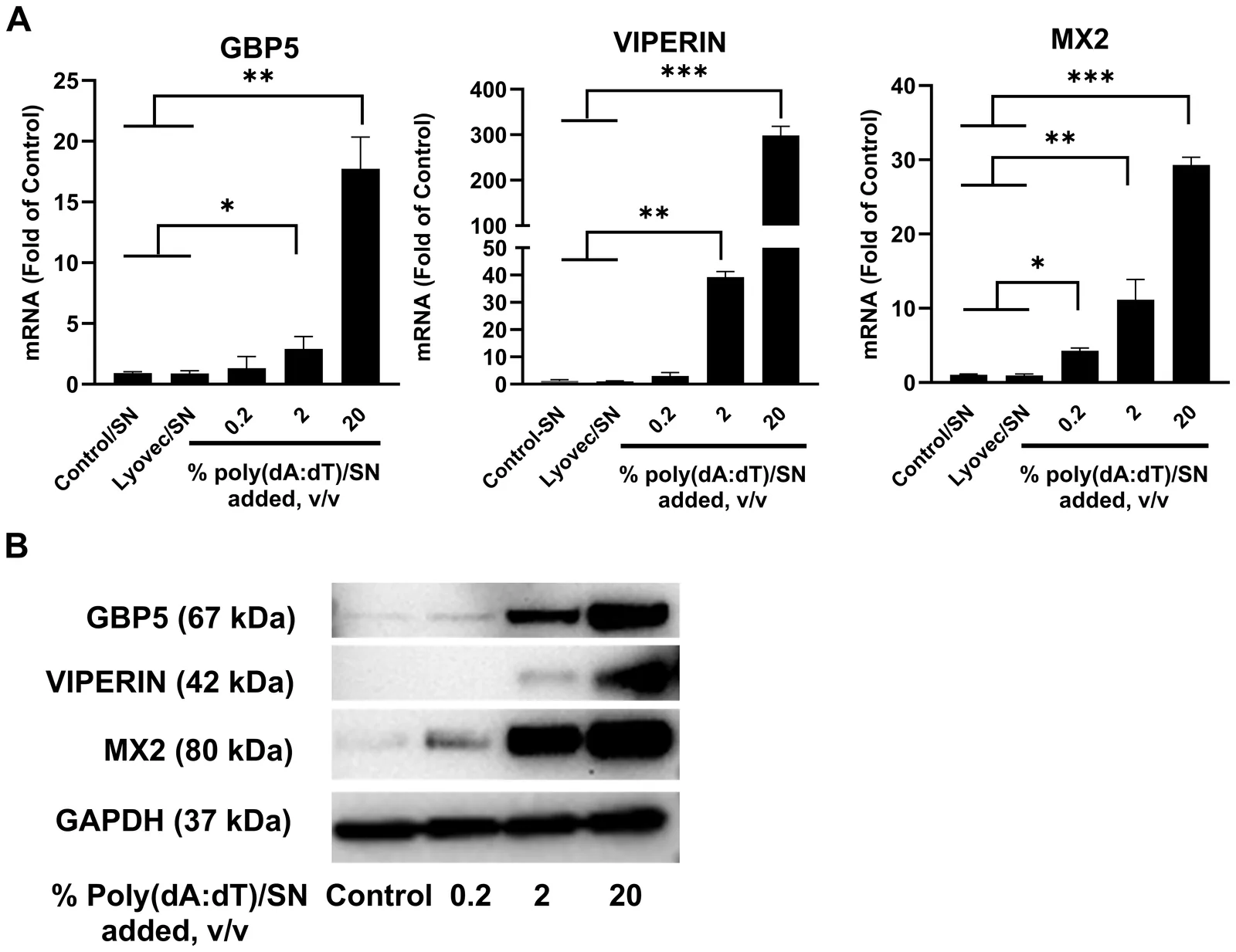
Supernatant (SN) from poly (dA:dT)-stimulated NCM460 cell cultures induces ISGs in macrophages. Macrophages were treated with different concentrations (0.2%, 2%, and 20%) conditioned SN from poly (dA:dT)-stimulated NCM460 cells, conditioned SN from Lyovec alone treated NCM460 cells, or conditioned SN from untreated NCM460 cells for 24 h. **(A)** Total RNA was extracted and subjected to quantitative RT-PCR for the expression of the indicated ISGs. Asterisks indicate that the differences between the indicated groups are statistically significant (*p < 0.05, **p < 0.01, ***p < 0.001). **(B)** Total cellular proteins were collected and subjected to Western blot with antibodies against VIPERIN, GBP5, and MX2. GAPDH was used as a loading protein control.

**Figure 6:**
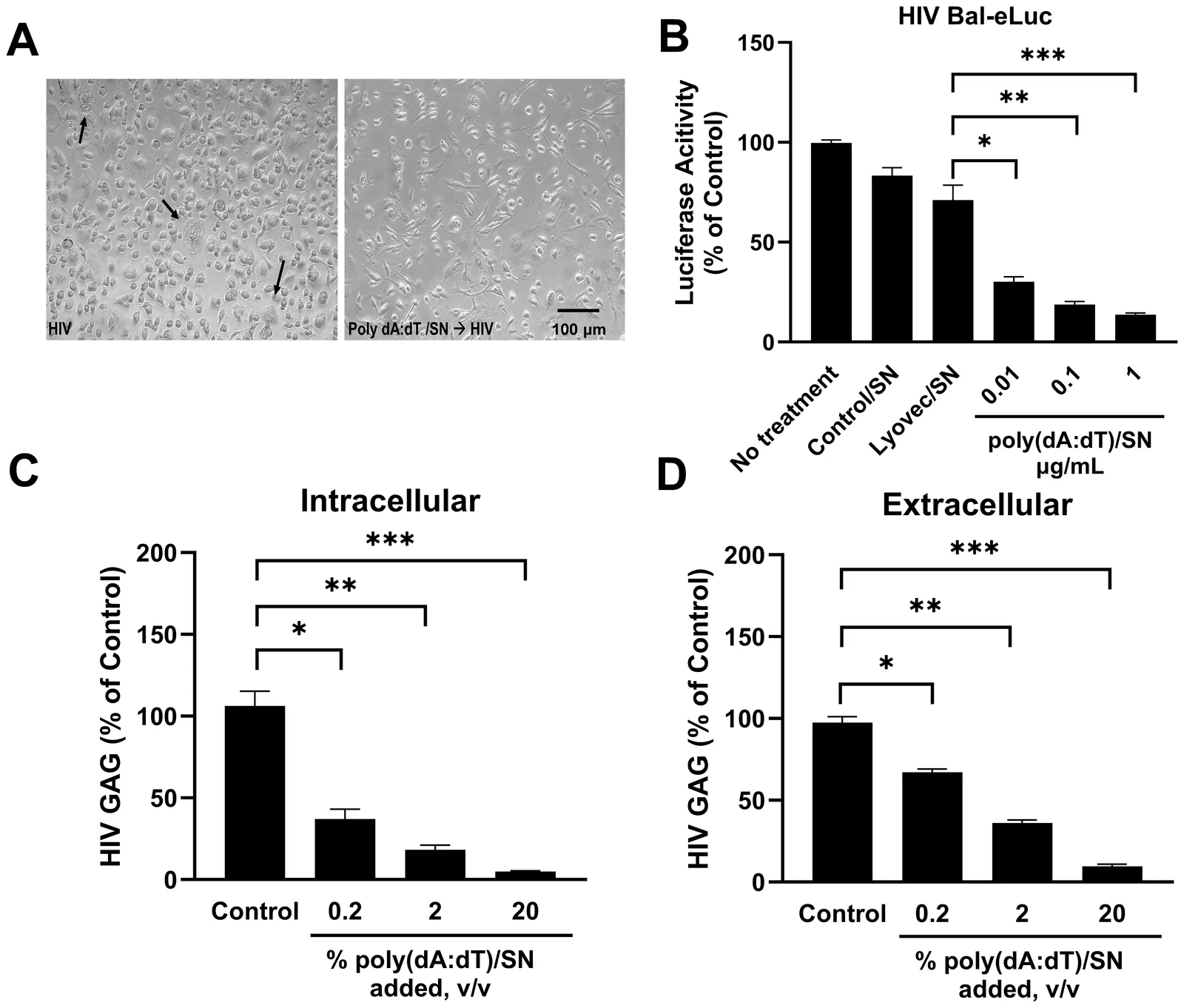
Antiviral activity of intestinal epithelial cell-derived soluble factors against HIV-1 infection in macrophages. NCM460 cells received poly (dA:dT) transfection at graded concentrations (0.01, 0.1, and 1μg/mL) for 6 h and followed by an 18h recovery period in fresh medium. The resulting conditioned supernatants, harvested at the 24h timepoint, were subsequently applied to primary human macrophage cultures to assess their impact on HIV-1 replication. **(A)** Morphological assessment of syncytia formation. HIV-1 Bal eLuc-infected macrophages were examined for cytopathic effects on day 6 following viral challenge. Light microscopy revealed striking differences in syncytium formation (indicated by arrows) between untreated controls and cells receiving epithelial-conditioned medium from poly (dA:dT)-activated cultures, with the latter showing marked reduction in multinucleated giant cell formation. **(B)** Quantitative luminescence-based analysis of viral replication. Primary macrophages underwent 24-hour pretreatment with epithelial-conditioned supernatants (diluted to 2% v/v) derived from NCM460 cultures stimulated with increasing poly (dA:dT) doses. Following overnight exposure to HIV-1 Bal-eLuc, cells were extensively washed (three PBS rinses) and maintained for six days before luciferase activity quantification. Control: No treatment. Triplicate measurements from three independent experimental series with different donors (mean ± SD) demonstrated dose-responsive viral suppression (statistical significance: * p < 0.05, ** p < 0.01, *** p < 0.001). **(C, D)** Dilution series analysis of antiviral potency. To determine the optimal concentration of epithelial-derived antiviral factors, macrophages were conditioned for 24 h with serial dilutions (0.2%, 2%, or 20% v/v) of supernatant from maximally stimulated (1 μg/ml poly dA:dT) NCM460 cultures before HIV-1 Bal strain inoculation. Six days post-infection, we performed comprehensive viral burden assessment through RT-PCR quantification of HIV gag transcripts in both cell-associated RNA **(C)** and cell-free supernatant **(D)**. Viral load measurements, normalized to untreated controls (defined as 100%), revealed concentration-dependent antiviral effects across three experimental replicates (mean ± SD; * p < 0.05, ** p < 0.01, *** p < 0.001).

## 4 Discussion

The gastrointestinal (GI) tract is a major site of HIV transmission, replication, and persistence, yet the contribution of intestinal epithelial cells (IECs) to antiviral immunity remains incompletely understood. Because IECs are in close contact with HIV virions and virus-infected immune cells, understanding their role in mucosal innate immunity is important. Using NCM460 cells, an established *in vitro* model derived from normal human colon epithelium (29, 31, 46, 47), our findings demonstrate that activation of cytosolic DNA sensing can induce antiviral responses and soluble factors capable of restricting HIV infection in primary human macrophages.

Previous studies have shown that activation of Toll-like receptor 3 (TLR3) in IECs induces antiviral activity against HIV (29); however, the potential contribution of cytosolic DNA sensing has been less well characterized. In this study, stimulation of NCM460 cells with poly(dA:dT), a synthetic double-stranded DNA analog, induced type I (IFN-β) and type III (IFN-λ1/2/3) interferons, as well as antiviral interferon-stimulated genes (ISGs), including ISG15, OAS-1, and Viperin. Poly(dA:dT) stimulation also increased the production of the CC chemokines MIP-1α and RANTES, which are natural ligands of the HIV co-receptor CCR5. These findings suggest that cytosolic DNA sensing in intestinal epithelial cells may contribute to antiviral innate immunity within the GI microenvironment.

Importantly, soluble factors released from poly(dA:dT)-stimulated NCM460 cells induced antiviral ISGs in primary human macrophages and significantly inhibited HIV infection. Several of the induced ISGs have established HIV-restrictive functions: ISG15 can interfere with viral assembly and release (47, 48), MX2 restricts HIV replication through post-entry mechanisms (48–50), and Viperin has broad antiviral activity involving interactions with viral and host pathways (51, 52). In addition, MIP-1α and RANTES can compete with HIV for CCR5 binding and thereby inhibit viral entry (43–45, 53). Thus, the combination of IEC-derived interferons, ISGs, and CC chemokines may contribute to an antiviral environment that limits HIV infection. However, the specific contributions of IFN signaling, CCR5-binding chemokines, and individual ISGs to the observed antiviral effect remain established through functional blocking and viral-entry studies.

These findings are particularly relevant because HIV has evolved multiple mechanisms to evade intracellular innate immune sensing, including suppression of cytosolic DNA sensing pathways in macrophages (30, 54–56). Key DNA sensing components, including cGAS and STING, are reduced in HIV-infected individuals even after antiretroviral therapy (57), and macrophages exhibit impaired interferon responses during infection (58–61). Our data suggest that paracrine signals released from activated intestinal epithelial cells may enhance innate antiviral responses in macrophages. This potential bystander effect supports a broader role for epithelial cell–macrophage crosstalk in shaping antiviral immunity within the GI microenvironment.

## 5 Limitations of this study

While we demonstrated that poly(dA:dT) activation of NCM460 cells induces soluble antiviral factors capable of suppressing HIV infection in macrophages, several limitations should be acknowledged. Our findings are consistent with, but do not definitively establish, the functional involvement of the cGAS–STING pathway, partly due to the difficulty of achieving complete knockdown of key DNA sensors, including cGAS, STING, and IFI16, using siRNA or shRNA. In addition, direct biochemical evidence of pathway activation, such as intracellular cGAMP levels and TBK1 or IRF3 phosphorylation, was not assessed. Although rigorous washing procedures were performed, potential carryover effects of transfection reagents or cellular stress-associated products cannot be completely excluded.

The specific contributions of IFN signaling and CC chemokines also remain to be established through functional receptor blockade or JAK inhibition. Direct measurement of macrophage surface CCR5 expression and viral-entry assays will be needed to distinguish inhibition of viral entry from post-entry restriction. Although HIV infection was evaluated using luciferase reporter assays and Gag RNA quantification, p24 capsid antigen was not measured. Finally, because our experiments used a single intestinal epithelial cell line (NCM460) and peripheral blood monocyte-derived macrophages, further validation using additional epithelial models, primary tissue-resident macrophages, and intestinal organoids will be important to better recapitulate mucosal physiology and confirm these findings *in vivo*.

## 6 Conclusion

The interaction between IECs and macrophages is an important component of mucosal immune defense (62–64). Our findings demonstrate that activation of cytosolic DNA sensing in NCM460 intestinal epithelial cells induces a coordinated antiviral response involving interferons, ISGs, and CCR5-binding chemokines. Soluble factors released from activated NCM460 cells enhanced antiviral responses in primary human macrophages and significantly restricted HIV infection. These findings support a potential role for epithelial cell-macrophage crosstalk in enhancing innate antiviral immunity within the GI microenvironment. Given the importance of macrophages as long-lived HIV reservoirs (65–70), further studies using additional epithelial models, primary GI-resident macrophages, intestinal organoids, and *in vivo* systems are warranted to validate these findings and determine their potential therapeutic relevance.

## Data Availability

The original contributions presented in the study are included in the article/**Supplementary Material**. Further inquiries can be directed to the corresponding author.
