# Supplementary material for: Activation of cytosolic DNA sensing in NCM460 intestinal epithelial cells induces antiviral factors that restrict HIV infection in macrophages
Source: Front Immunol. 2026 Sep 30;17:1919977. doi: 10.3389/fimmu.2026.1919977 (PMC13661426; doi:10.3389/fimmu.2026.1919977)
Supplement: Supplementary file 1 [file DataSheet1.pdf]

Supplemental Figure 1

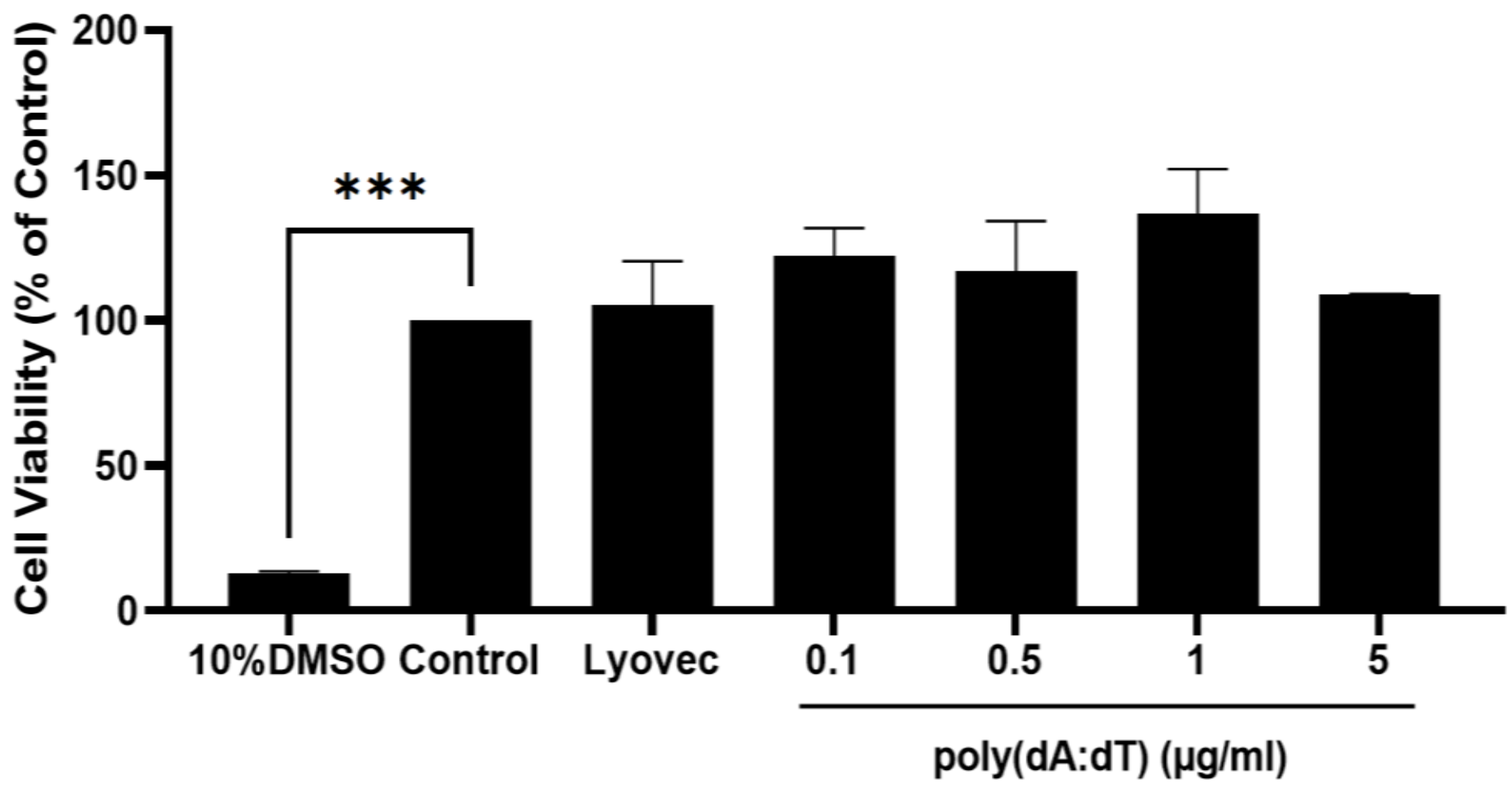

Supplemental Figure 1: Effect of poly (dA:dT) on the cell viability of human IECs. NCM460 cells were treated with poly (dA:dT) at the indicated concentrations of for 48 h. The cell viability was assessed by MTS assay. DMSO (10%) was used as a positive control. Data are shown as the absorbance (490 nm) relative to untreated control, which is defined as 100. The results shown were obtained as mean ± SD from three independent experiments with triplicate wells (***$p < 0.001$).

Supplemental Table 1. Primers for Real-time PCR

Gene	Forward (5′–3′)	Reverse (5′–3′)
**cGAS**	GGGAGCCCTGCTGTAACACTTCTTAT	CCTTTGCATGCTTGGGTACAAGGT
**STING**	ACTGTGGGGTGCCTGATAAC	TGGCAAACAAAGTCTGCAAG
**IFI16**	CCGTTCATGACCAGCATAGG	TCAGTCTTGGTTTCAACGTGGT
**IFR1**	TGAAGCTACAACAGATGAGG	AGTAGGTACCCCTTCCCATC
**IFR7**	TGGTCCTGGTGAAGCTGGAA	GATGTCGTCATAGAGGCTGTTGG
**IFR9**	GCATCAGGCAGGGCACGCTGCACC	GCCTGCATGTTTCCAGGGAATCCG
**IFN-β**	GCCGCATTGACCATCTATGAGA	GAGATC TTCAGTTT CGGAGGTAAC
**IFNλ1**	CTTCCAAGCCCACCCC AACT	GGCCTCCAGGACCTTCAGC-3
**IFNλ2/3**	TTTAAGAGGGCCAAAGATGC	TGGGCTGAGGCTGGATACAG
**MIP-1α**	GCTGACTACTTTGAGACGAGC	CCAGTCCATAGAAGAGGTAGC
**RANTES**	CTGCATCTGCCTCCCCATA	GCGGGCAATGTAGGCAAA
**GBP5**	TGGCAAATCCTACCTGATGA	CCATATCCAAATTCCCTTGG
**VIPERIN**	TGGGTGCTTACACCTGCTG	TGAAGTGATAGTTGACGCTGGT
**MX2**	CAGCCACCACCAGGA AACA	TTCTGCTCGTACTGGCTGTACAG
**ISG15**	GGCTGGGAGCTGACGGTGAAG	GCTCCGCCCGCCAGGCTCTGT
**OAS1**	AGAAGGCAGCTCACGAAACC	CCACCACCCAAGTTTCCTGTA
**GAPDH**	GGTGGTCTCCTCTGACTTCAACA	GTTGCTGTAGCCAAATTCGTTGT
